# CDC Updates Guidelines for Children’s Lead Exposure

**DOI:** 10.1289/ehp.120-a268

**Published:** 2012-07-02

**Authors:** Kellyn S. Betts

**Affiliations:** For more than a dozen years Kellyn S. Betts has written about environmental contaminants, hazards, and technology for solving environmental problems for publications including *EHP* and *Environmental Science & Technology*.

In response to the growing body of scientific evidence about effects of exposure to low levels of lead,^1^ the U.S. Centers for Disease Control and Prevention (CDC) has announced it is changing its guidelines for children’s exposure to the toxic metal, reducing by half the blood lead level at which intervention is recommended.^2^ According to CDC spokesman Jay Dempsey, the agency estimates the change will increase the number of affected children from fewer than 100,000 to 372,979. Recommended intervention involves identifying and removing sources of lead exposure and follow-up blood lead monitoring.

Since 1991 the CDC’s “level of concern” for lead in children’s blood had been set at 10 µg/dL. At the recommendation of its Advisory Committee on Childhood Lead Poisoning Prevention, the agency is dispensing with the use of a static number. Instead, its new reference value of 5 µg/dL is based on the 97.5th percentile of blood lead levels in U.S. children aged 1–5 years, as measured by the agency’s National Health and Nutrition Examination Survey (NHANES). The reference value will be updated every four years based on the two most recent iterations of the NHANES.^2^

The CDC has moved away from the “level of concern” terminology because it “gives a false sense of security to people” whose children’s blood lead fell below the cutoff, says Christopher Portier, director of the agency’s National Center for Environmental Health. The agency and its advisory committee can find no evidence that there is a safe level of lead exposure for children, he says.

**Figure:**
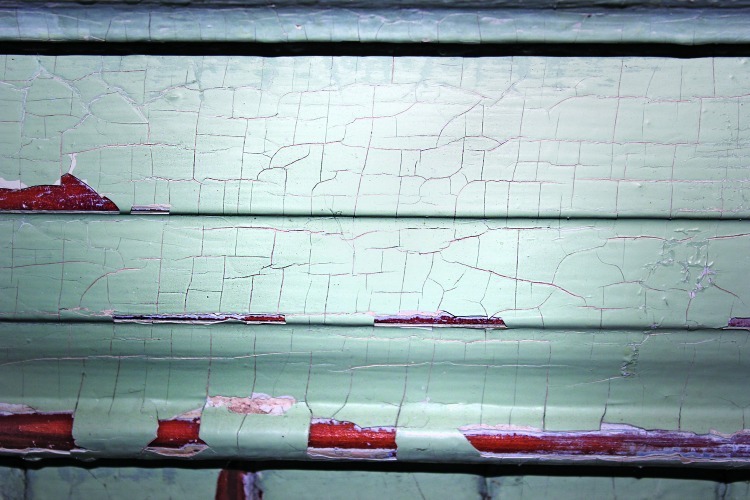
Lead-based paint remains the most important source of lead poisoning for U.S. children.^1^ Despite the great reductions in exposure that followed the removal of lead from gasoline, an estimated 37.1 million U.S. homes still contain lead paint.^6^ Ingestion of paint chips or (more commonly) of the contaminated dust that forms when lead paint erodes is the usual route of pediatric exposure. The “checking” pattern of surface cracks seen in this image is typical of older lead-based paint, although the absence of a checking pattern does not guarantee that a surface is lead-free, because older lead-based paint may be painted over. A definitive diagnosis requires X-ray fluorescence analysis of each painted surface, especially those within reach of young children such as window sills and railings. Such analysis is strongly recommended for any U.S. home built before 1978, the year residential use of lead-based paint was banned. Testing should be undertaken by an inspector certified by the EPA or the local health department. Philip J. Landrigan, MD, MSc, and Charles H. Kellner, MD

A host of studies now link exposure to lower levels of lead with behavior problems, attention deficit/hyperactivity disorder, and adverse cardiovascular, immunological, and endocrine effects, says Bruce Lanphear of Simon Fraser University. Lanphear led a 2005 study in which seven different longitudinal cohorts from multiple countries reported very similar decreases in intelligence in association with blood lead levels below 10 µg/dL.^3^ In June 2012 the National Toxicology Program concluded that adverse health effects occur at levels below even 5 µg/dL.^4^

Homes can be a major source of lead exposure, and the CDC is focused on primary prevention, says Mary Jean Brown, chief of the agency’s Lead Poisoning Prevention Branch. “We have to develop an agenda that controls or eliminates lead hazards before children are poisoned,” she says. The CDC is working with the U.S. Department of Housing and Urban Development and the U.S. Environmental Protection Agency to identify communities where high proportions of children have elevated lead levels and to take actions to remediate the sources of exposure, she says.

“For most children, the majority of their exposure is from household dust that is a consequence of the decay of old lead-based paint,” says Michael Weitzman, a professor of environmental medicine and pediatrics at New York University’s Langone Medical Center. Old windows are a major source of exposure; another is renovating older homes without taking proper precautions, according to the nonprofit National Center for Healthy Housing (NCHH).

However, less is known about how to clean up these sources of lead. Lanphear points to the need for effective, evidence-based methods for reducing lead in house dust, soil, water, and consumer products. He is working on a study that he says demonstrates the efficacy of lead hazard controls to reduce children’s blood lead levels.

Despite dramatic cuts to the CDC’s Healthy Homes/Lead Poisoning Prevention Program in fiscal year 2012,^5^ the May 2012 announcement of the new reference value constitutes a promise of action, Portier says. He says the agency will develop educational materials to explain to health departments, pediatricians, nongovernmental organizations, and other groups what the change means and how to act on it. However, 80% of the program budget supported state and local lead-poisoning prevention programs that will no longer be funded, says Dempsey. Portier says he is beginning to see many of these programs wind down.

No matter how the new guidelines are ultimately implemented, NCHH executive director Rebecca Morley hopes more families will start to understand that even very low lead exposures can cause harm. She says the decision will prompt follow-up educational activities to help ensure that “parents are going to begin to get the information that they deserve.”
